# Power estimation and sample size determination for replication studies of genome-wide association studies

**DOI:** 10.1186/s12864-015-2296-4

**Published:** 2016-01-11

**Authors:** Wei Jiang, Weichuan Yu

**Affiliations:** Department of Electronic and Computer Engineering, The Hong Kong University of Science and Technology, Clear Water Bay, Kowloon, Hong Kong, China

**Keywords:** Replication study, Power, Empirical Bayes

## Abstract

**Background:**

Replication study is a commonly used verification method to filter out false positives in genome-wide association studies (GWAS). If an association can be confirmed in a replication study, it will have a high confidence to be true positive. To design a replication study, traditional approaches calculate power by treating replication study as another independent primary study. These approaches do not use the information given by primary study. Besides, they need to specify a minimum detectable effect size, which may be subjective. One may think to replace the minimum effect size with the observed effect sizes in the power calculation. However, this approach will make the designed replication study underpowered since we are only interested in the positive associations from the primary study and the problem of the “winner’s curse” will occur.

**Results:**

An Empirical Bayes (EB) based method is proposed to estimate the power of replication study for each association. The corresponding credible interval is estimated in the proposed approach. Simulation experiments show that our method is better than other plug-in based estimators in terms of overcoming the winner’s curse and providing higher estimation accuracy. The coverage probability of given credible interval is well-calibrated in the simulation experiments. Weighted average method is used to estimate the average power of all underlying true associations. This is used to determine the sample size of replication study. Sample sizes are estimated on 6 diseases from Wellcome Trust Case Control Consortium (WTCCC) using our method. They are higher than sample sizes estimated by plugging observed effect sizes in power calculation.

**Conclusions:**

Our new method can objectively determine replication study’s sample size by using information extracted from primary study. Also the winner’s curse is alleviated. Thus, it is a better choice when designing replication studies of GWAS. The R-package is available at: http://bioinformatics.ust.hk/RPower.html.

## Background

Genome-wide association studies (GWAS) are widely used to identify susceptibility variants of common diseases. Commonly, single nucleotide polymorphisms (SNPs) are genotyped across the whole genome in different individuals, and statistical methods are used to detect the associations between SNPs and disease status. According to the summary of GWAS catalog ([1], accessed [2015.05.28]), about 2000 GWAS reports related to 756 diseases/traits have been published so far, from which 14,609 associations show genome-wide significance (*p*-value ≤5×10^−8^). More and more associations will be discovered from GWAS.

The basic statistical method used in GWAS analysis is hypothesis testing [2]. The possibilities of false positives cannot be completely removed in the analysis. Hence, all findings from GWAS need to be verified. Replication study is a commonly used approach to verifying positive findings [3, 4]. If an association between one specific SNP and a certain disease has been identified in the primary study and confirmed in the replication study, we usually treat this association as true positive with a high confidence. If an association identified in the primary study cannot be confirmed in the replication study, we often suspect that it is a false positive.

The power of replication study is crucial in this validation process. If the replication study is underpowered, then the positive findings will have a low chance to be replicated. It’s essential to design a replication study with enough statistical power.

How to estimate the power of a replication study in the design phase?

Traditionally, a replication study is regarded as another independent primary study. Thus, the same power calculation in the original primary study is used. For the associations identified in the primary study, a minimum effect size needs to be specified. Then, the underlying alternative distribution of test statistics is assumed to have specified effect size. The major limitation of this traditional power calculation method is that the specification of the effect size is subjective and may cause bias. Besides, no information from primary study has really been used.

One may think to plug the observed effect sizes from the primary study in the power calculation of the replication study. This power estimation approach doesn’t need to specify any parameters. Since only significant associations are considered in the replication study, the observed effect sizes for those associations will tend to be overestimated [5]. This phenomenon is known as the “winner’s curse” [6], which makes the estimated powers tend to have higher values.

A lot of methods have been proposed to overcome the winner’s curse in effect size estimation. An incomplete list includes conditional maximum likelihood estimation (CMLE, [7–9]), bootstrap [10], full Bayesian method [11] and Empirical Bayes method (EB, [12]). Since power function is usually not a linear function of effect size, the estimators obtained by simply plugging those bias-corrected effect sizes in power calculation may not achieve the best performance.

**Fig. 1:**
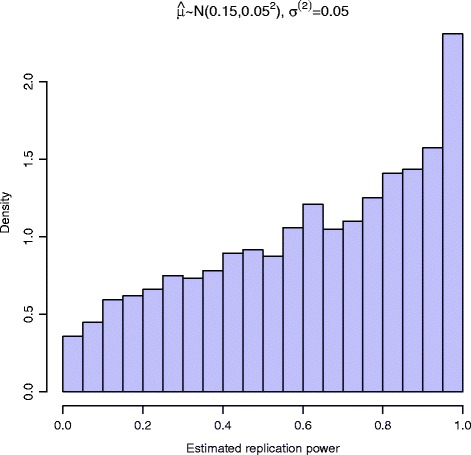
When *log*-odds ratio is normally distributed, the power of replication study possesses non-normality. Assume the effect size follows normal distribution *N*(0.15,0.05^2^). The standard error of *log*-odds ratio is *σ* ^(2)^=0.05 in the replication study. The significance level of the replication study is *α* ^(2)^=5×10^−3^. The histogram of the power in the replication study possesses non-normality

Moreover, there are two other challenges in designing replication study:

1. Due to the nonlinear nature and restricted range (limited to [ 0,1]) of power function, the distribution of power is usually non-normal when effect size is normally distributed (illustrated in Fig. 1). The interval estimation of the power should consider the non-normality.
2. Since the power values of different associations in the primary study are different, a summary value is needed to determine the sample size of replication study.

This paper aims at addressing the above challenges. Our contributions are listed in the following:

1. For each association identified from the primary study, an EB based method is proposed to estimate its power in the replication study.
2. Due to the non-normality of the estimated power and the inaccuracy of the hyperparameters estimation, a novel interval estimation method combining Monte Carlo sampling and Bootstrap is proposed to estimate the corresponding credible interval of each association’s power in the replication study.
3. The average power of the discovered true associations is used for determining the sample size of replication study. An weighted average method is proposed to estimate the average power. Our proposed interval estimation method can also be used to construct the credible interval of the average power.
4. Only the summary statistics of the primary study are needed when using our proposed method to design a replication study. This feature is helpful since summary statistics are more accessible than individual-level genotype data due to the privacy issue and other constraints.

The rest of this paper is organized as follows. In section ‘Methods’, we will introduce the Bayesian framework to estimate the power of replication studies. We will prove that Bayesian predictive power is immune to the winner’s curse. Then we will present how to estimate the power with two-component mixture prior under the Bayesian framework. We will also give the details about estimation of hyperparameters, interval estimation and the estimation of average power. In section ‘Results and discussion’, we will first use simulation results to demonstrate that our EB based method is better than other plug-in based estimators in terms of overcoming the winner’s curse and providing higher estimation accuracy. We will also demonstrate that the coverage probability of given credible interval is well-calibrated. Then we will show the sample sizes determined to replicate findings of 6 diseases from Wellcome Trust Case Control Consortium (WTCCC) [13], which are much higher than the sample sizes estimated by plugging observed effect sizes in the power calculation formula. The increased sample sizes are reasonable due to the winner’s curse. In the same section, we will discuss limitations of current modeling and estimation approach. Section ‘Conclusions’ concludes the paper.

## Methods

We use parenthesized superscript “ (*j*)” to denote primary study (*j*=1) and replication study (*j*=2). For example, we denote the sample size in the primary study as *n*^(1)^. The sample size in the control group and case group are $n_{0}^{(1)}$ and $n_{1}^{(1)}$, respectively. The total number of SNPs genotyped in the primary study is *m*. Among those genotyped SNPs, the proportion of the SNPs having no association with the disease (null SNPs) is *π*_0_(0≤*π*_0_≤1).

In both the primary study and the replication study, a contingency table can be created as in Table 1 for each genotyped SNP. With the contingency table, the logarithm of the observed odds ratio reads:

(1) $$ \widehat{\mu}^{(j)}=\log n_{00}^{(j)}-\log n_{01}^{(j)}-\log n_{10}^{(j)}+\log n_{11}^{(j)}.  $$

**Table 1:** Allele based contingency table of one SNP in primary/replication study. Please see the main text for explanation of the notations

	Non-effect allele	Effect allele	Total
Control	$n_{00}^{(j)}$	$n_{01}^{(j)}$	$2n_{0}^{(j)}$
Case	$n_{10}^{(j)}$	$n_{11}^{(j)}$	$2n_{1}^{(j)}$
Total	$n_{00}^{(j)}+n_{10}^{(j)}$	$n_{01}^{(j)}+n_{11}^{(j)}$	2*n* ^(*j*)^

The true value of the log odds ratio *μ* is usually unknown. The asymptotic standard error of $\widehat {\mu }^{(j)}$ can be approximated using Woolf’s method [14],

(2) $$ \sigma^{(j)}\approx \sqrt{\frac{1}{n_{00}^{(j)}}+\frac{1}{n_{01}^{(j)}}+ \frac{1}{n_{10}^{(j)}}+\frac{1}{n_{11}^{(j)}}}.  $$

To test whether there is an association between the SNP and the disease, two hypotheses are set up:

(3) $$ \mathcal{H}_{0}:\ \mu=0,~\text{vs.}~ \mathcal{H}_{1}:\ \mu\neq 0.  $$

Wald test can be used to examine whether the null hypothesis should be rejected. The test statistic is $z^{(j)}=\widehat {\mu }^{(j)}/\sigma ^{(j)}$. The significance levels in the primary study and the replication study are fixed to *α*_1_ and *α*_2_, respectively.

Two-sided test is used in primary study. The rejection region is $|Z^{(1)}|>z_{\alpha _{1}/2}$ (We use uppercase letter to indicate a random variable), where *z*_*u*_(0≤*u*≤0.5) is the upper *u* quantile of the standard normal distribution *N*(0,1). For a replicated association, the test statistics in two studies should be consistent with the same sign. Hence, the test can be regarded as one-sided test in replication study. The rejection region is $sgn\left (z^{(1)}\right)Z^{(2)}>z_{\alpha _{2}}$, where the sign function is

(4) $$ sgn(x)=\left\{ \begin{array}{ll} 1 & \text{if}~ x>0 \\ 0 & \text{if}~ x=0\\ -1 & \text{if}~ x<0 \end{array} \right.\,.  $$

### Bayesian predictive power

For an association identified in primary study, the power function in replication study is defined as

(5) $$ \begin{aligned} \beta^{(2)}(\mu)&=P\left(sgn\left(z^{(1)}\right)Z^{(2)}>z_{\alpha_{2}} \big| \mu, z^{(1)}, \mathcal{H}_{1}\right),\\ &\quad\text{where}~ |z^{(1)}|>z_{\alpha_{1}/2}. \end{aligned}   $$

A traditional power calculation method needs to specify a minimum detectable effect size *μ*_*min*_ first. Then, the power of replication study is *β*^(2)^(*μ*_*min*_). Consequently, the power can be used to determine the sample size.

To incorporate information from primary study, the post-hoc method estimates the power of each association by plugging the observed effect size in Eq. (5), i.e. $\beta ^{(2)}\left (\widehat {\mu }^{(1)}\right)$. This approach is widely criticized for the reason of the winner’s curse. The estimated power is biased upward since only significant associations are selected in the replication study. To address this problem, a lot of methods have been proposed to overcome the winner’s curse in effect size estimation [7–12]. Conditional maximum likelihood estimation (CMLE) is the most commonly used type [7–9]. In CMLE, the effect size is estimated by maximizing the likelihood conditioning on rejected region, i.e.

(6) $$ \widehat{\mu}^{(1)}_{CMLE}=arg \max_{\mu} P\left(z^{(1)}\big| \mu, |Z^{(1)}|>z_{\alpha_{1}/2}, \mathcal{H}_{1}\right).  $$

Please notice that, although the selection bias can be reduced using estimator which can adjust estimated effect size, no unbiased estimator exists [11]. With estimated effect size, the power of replication study can be obtained by using $\beta ^{(2)}\left (\widehat {\mu }_{\textit {CMLE}}^{(1)}\right)$. The plug-in based power estimator is not optimized in terms of minimizing Bayes risk.

The Bayes risk averages the loss function over both sample space and parameter space. In terms of overcoming the winner’s curse, the Bayes risk $R(\widehat {\theta })$ should be defined conditioning on rejected region and alternative hypothesis,

(7) $$ R(\widehat{\theta})=E_{\mu, Z^{(1)}}\left(\left(\widehat{\theta}-\beta^{(2)}(\mu)\right)^{2}\big||Z^{(1)}|>z_{\alpha_{1}/2}, \mathcal{H}_{1}\right),  $$

where $\widehat {\theta }$ is the power estimator of replication study. Inspired by the proof in [12], we can show that Bayesian predictive power *η*^(2)^ [15] is the estimator minimizing $R(\widehat {\theta })$ (please see Appendix for detail). The Bayesian predictive power reads

(8) $$ \begin{aligned} \eta^{(2)}&=P\left(sgn\left(z^{(1)}\right)Z^{(2)}>z_{\alpha_{2}}\big| z^{(1)}, \mathcal{H}_{1}\right)\\ &=E_{\mu}\left(\beta^{(2)}(\mu)\big| z^{(1)}, \mathcal{H}_{1}\right), \end{aligned}  $$

which takes the average of all power function values among all possible *μ* values given observed *z*^(1)^. We will provide a detailed formula of the Bayesian predictive power under one specific prior in the following subsection.

### Two-component mixture prior

In each study, the observed log odds ratio $\widehat {\mu }^{(j)}$ asymptotically follows normal distribution *N*(*μ*,(*σ*^(*j*)^)^2^). The underlying true value of the effect size *μ* is often unknown. It is widely suspected that a large proportion of SNPs with small effect sizes are associated with complex diseases [16, 17]. We use Gaussian prior to depict this pattern of the associated SNPs. For all SNPs, we use the following two-component mixture prior to describe their effect sizes:

(9) $$ \mu\sim \pi_{0} \delta_{0}+(1-\pi_{0}) N\left(0, {\sigma_{0}^{2}}\right),   $$

where *δ*_0_ is the distribution with point mass on zero and ${\sigma _{0}^{2}}$ is the variance of the effect sizes in associated SNPs.

With this prior, the posterior distribution of effect size *μ* under $\mathcal {H}_{1}$ is

(10) $$ \begin{aligned} \left(\mu|z^{(1)},\mathcal{H}_{1}\right)&\sim N\left(\lambda \widehat{\mu}^{(1)},\lambda \left(\sigma^{(1)}\right)^{2}\right),\\ \text{where}~ \lambda&=\frac{1}{1+\left(\sigma^{(1)}/\sigma_{0}\right)^{2}}. \end{aligned}   $$

The Bayesian predictive power of replication study is (Detail in the Appendix):

(11) $$ \eta^{(2)}= \Phi\left(\frac{sgn\left(z^{(1)}\right)z^{*}-z_{\alpha_{2}}}{\sigma^{*}}\right),   $$

where $z^{*}=\lambda \widehat {\mu }^{(1)}/\sigma ^{(2)}$, $\sigma ^{*}=\sqrt {1+\lambda \left (\frac {\sigma ^{(1)}}{\sigma ^{(2)}}\right)^{2}}$ and *Φ*(*x*) is the cumulative density function (cdf) of *N*(0,1). By substituting observed allele frequencies from the primary study into Woolf’s method, *σ*^(2)^ can be approximated as

(12) $$ \sigma^{(2)}\approx \sqrt{\frac{n_{0}^{(1)}}{n_{0}^{(2)}}\left(\frac{1}{n_{00}^{(1)}}+\frac{1}{n_{01}^{(1)}}\right)+ \frac{n_{1}^{(1)}}{n_{1}^{(2)}}\left(\frac{1}{n_{10}^{(1)}}+\frac{1}{n_{11}^{(1)}}\right)}.  $$

There is an unknown hyperparameter ${\sigma _{0}^{2}}$ in the calculation of Bayesian predictive power. In the following subsection, we will present how to estimate ${\sigma _{0}^{2}}$ with Empirical Bayes approach.

### Hyperparameter ${\sigma _{0}^{2}}$

In Empirical Bayes’ thinking, we can estimate ${\sigma _{0}^{2}}$ by taking advantage of the shared structure of the effect size’s distribution among all SNPs, which can be seen from Eq. (9). The estimator of ${\sigma _{0}^{2}}$ is (see Appendix for detail):

(13) $$ \widehat{\sigma}_{0}^{2}\,=\,\max\!\left(\!0, \!\left(\!\frac{\sum_{i=1}^{m} \left(z^{(1)}_{i}\right)^{2}\,-\,m\pi_{0}}{\left(1\,-\,\pi_{0}\right)}\,-\,m\right)\!/\!\sum_{i=1}^{m} \left(1/\sigma^{(1)}_{i}\!\right)^{2}\! \right).   $$

There are two extreme cases in the above estimation:

1. If the null hypothesis is valid, then all SNPs follow a standard normal distribution with variance equal to one. When $\frac {1}{m}\sum _{i=1}^{m} \left (z^{(1)}_{i}\right)^{2}\leq 1$, i.e., the sample variance is no bigger than one as in the null hypothesis case, we will have $\widehat {\sigma }_{0}^{2}=0$. In this case, the result of our EB based power estimation method will degenerate to type I error rate, which is the probability that the identified association can be replicated even when the association doesn’t exist.
2. When $\frac {1}{m}\sum _{i=1}^{m} \left (z^{(1)}_{i}\right)^{2}>1$ but *π*_0_=1, we will have $\widehat {\sigma }_{0}^{2}=+\infty $. In this case, the above shrinkage coefficient will degenerate to *λ*=1. The shrinkage effect in our EB based method will disappear.

Noticed that there is another unknown hyperparameter *π*_0_ in calculating $\widehat {\sigma }_{0}^{2}$. The estimation of the proportion of true null hypotheses *π*_0_ has been extensively studied [18–20]. Here we just choose Storey’s method [18] for the simplicity of implementation. Let’s denote the number of SNPs with *p*-value >*γ* as *m*_+_(*γ*) in the primary study. Then *π*_0_ can be estimated by using

(14) $$\begin{array}{@{}rcl@{}} \widehat{\pi}_{0}=\frac{m_{+}(\gamma)}{m(1-\gamma)}. \end{array} $$

There is a bias-variance tradeoff in tuning *γ*. An automatic procedure is proposed in [18] without tuning *γ*: A natural cubic spline will fit to evaluated values with different *γ*, then $\widehat {\pi }_{0}$ is the spline’s value at *γ*=1.

$\widehat {\sigma }_{0}^{2}$ can be calculated by plugging $\widehat {\pi }_{0}$ in Eq. (13). By plugging $\widehat {\sigma }_{0}^{2}$ into Eq. (11), an EB based estimator of the replication study’s power can be obtained, which is denoted as $\widehat {\eta }^{(2)}_{\textit {EB}}$. The corresponding credible interval can be constructed, which is presented in the following subsection.

### Credible interval

From Eq. (10), the posterior distribution of log odds ratio *μ* under alternative hypothesis $\mathcal {H}_{1}$ is a normal distribution. Figure 1 shows the histogram of power values when *μ* is normally distributed. The shape of the histogram indicates the non-normality of the calculated power. Hence, the asymptotic approach based on normal distribution theory is not appropriate in the interval estimation of the replication study’s power. The construction of the credible interval should consider the non-normality. We propose to use Monte Carlo sampling to construct the credible interval of *β*^(2)^(*μ*). The credible interval is constructed with known hyperparameters ${\sigma _{0}^{2}}$. Since estimation error will occur in estimating ${\sigma _{0}^{2}}$, the constructed credible interval will have smaller coverage probability than nominal level. To incorporate the variance of the estimator $\widehat {\sigma }_{0}^{2}$, a method combining Monte Carlo sampling and Bootstrap is proposed. The test statistics from the primary study *z*^(1)^ will be resampled *N*_1_ times with replacement. For each run, ${\sigma _{0}^{2}}$ is re-estimated. Monte Carlo sampling is used to generate *N*_2_ power values with each re-estimated ${\sigma _{0}^{2}}$. The credible interval is constructed among all *N*_1_*N*_2_ sampled power values.

### Average power

Usually, multiple associations are identified in primary study. To design a replication study, a summarized value reflecting the average power of all associated SNPs is needed. A direct thinking is to average power among the identified SNPs with underlying true associations, which reads

(15) $$ \bar{\beta}^{(2)}(\mu)=\frac{1}{|S|}\sum_{i\in S} \beta^{(2)}(\mu_{i}),  $$

where *S* is the index set of the associated SNPs identified from primary study and |*S*| is the cardinality of *S*. The subscript *i* means that the quantity is evaluated for SNP *i*.

Since the index set *S* is unknown, we propose to use weighted average of the estimated powers $\widehat {\eta }_{\textit {EB}}^{(2)}$. The local true discovery rate (*ltdr*) of each SNP is the posterior probability of being associated SNP given observed statistics, which is complementary to local false discovery rate [21]. We use *ltdr* as weight in the estimation. The estimated average power is

(16) $$ \bar{\eta}^{(2)}_{EB}=\frac{\sum_{i\in T} {ltdr}_{i}^{(1)}\widehat{\eta}_{EB,i}^{(2)}}{\sum_{i \in T} {ltdr}_{i}^{(1)}},  $$

where *T* is the index set of the SNPs identified from the primary study. The local true discovery rate of the primary study can be calculated as (See Appendix for detail):

(17) $$ ltdr^{(1)}= \frac{\pi_{1} \phi \left(z^{(1)} /\sqrt{1+(\widehat{\sigma}_{0}/\sigma^{(1)})^{2}}\right)} {\pi_{0} \phi\left(z^{(1)}\right) +\pi_{1} \phi \left(z^{(1)} /\sqrt{1+\left(\widehat{\sigma}_{0}/\sigma^{(1)}\right)^{2}}\right)},   $$

where *ϕ*(*x*) is the probability density function (pdf) of *N*(0,1).

By setting the estimated average power larger than a threshold, e.g. $\bar {\eta }^{(2)}_{\textit {EB}}>80\,\%$, the sample size of replication study can be determined.

### Credible interval of the average power

The proposed interval estimation method can also be used to construct the credible interval of the average power. We resample the test statistics from the primary study *N*_1_ times. In each run, a re-estimated variance of the effect sizes in the non-null SNPs $\widehat {\sigma }_{0}^{2}$ can be obtained. For a fixed $\widehat {\sigma }_{0}^{2}$ value, we first calculate the local true discovery rate of the primary study *ltdr*^(1)^ with Eq. (17) for each association. Then Monte Carlo sampling is used to generate *N*_2_ sets of the power values, in each of which there are power values of the replication study for all associations identified from the primary study. In each set, an average power can be obtained by taking weighted average of those generated power values among all associations. Hence, *N*_2_ average power values can be generated in each run. The credible interval of average power can be constructed among all *N*_1_*N*_2_ sampled average power values.

## Results and discussion

### Simulation experiments

The following questions are examined using simulation experiments:

1. Can EB based power estimator $\widehat {\eta }_{\textit {EB}}^{(2)}$ perform well in terms of overcoming the winner’s curse?
2. Can $\widehat {\eta }_{\textit {EB}}^{(2)}$ estimate power accurately?
3. Is the corresponding credible interval well-calibrated?
4. Can weighted average estimator $\bar {\eta }_{\textit {EB}}^{(2)}$ estimate average power $\bar {\beta }^{(2)}(\mu)$ accurately?

In simulation experiments, sample sizes are $n^{(1)}_{0}=n^{(1)}_{1}=1000$ and ${n^{(2)}_{0}=n^{(2)}_{1}=500}$ in primary study and replication study, respectively. The number of simulated SNPs is *m*=1×10^4^. For each SNP, its minor allele frequency is uniformly distributed as *U*(0.05,0.5). Their effect sizes are generated from the following distribution:

(18) $$ \mu\sim 0.9\delta_{0}+0.1N(0,0.04).  $$

For our hypothetical disease, its prevalence is 1 *%*. To test the marginal association between SNPs and the disease, *log*-odds ratio test is used. The significance levels are *α*_1_=5×10^−5^ and *α*_2_=5×10^−3^ in primary study and replication study, respectively.

**Fig. 2:**
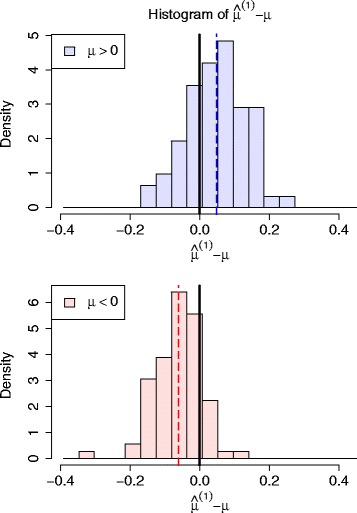
The winner’s curse exists in the estimation of effect size for SNPs identified from the primary study. The histograms of the estimation error $\widehat {\mu }^{(1)}-\mu $ for positive effect SNPs (SNPs with *log*-odds ratio greater than 0) and negative effect SNPs (SNPs with *log*-odds ratio smaller than 0) are plotted separately. The mean values of the estimation error is drawn with vertical dashed lines. From the figure, it can be concluded that the magnitude of the observed effect sizes $\hat {\mu }^{(1)}$ tend to be larger than the magnitude of their true effect. The observed effect sizes tend to be exaggerated

Figure 2 shows the histogram of the differences between observed effect sizes $\widehat {\mu }^{(1)}$ and their underlying true values *μ* for identified true associations. We plotted separatively for the associations with positive effect (*μ*>0) and negative effects (*μ*<0). For the associations with positive effect, the mean value of the estimated effect sizes is larger than the mean value of true effect sizes. On the contrary, the values of the estimated effect sizes tend to be smaller than their true values for the associations with negative effect. In both of these two cases, the magnitude of the observed effect sizes tend to be exaggerated, which indicates that the winner’s curse generally exists in the associations identified from primary study.

**Fig. 3:**
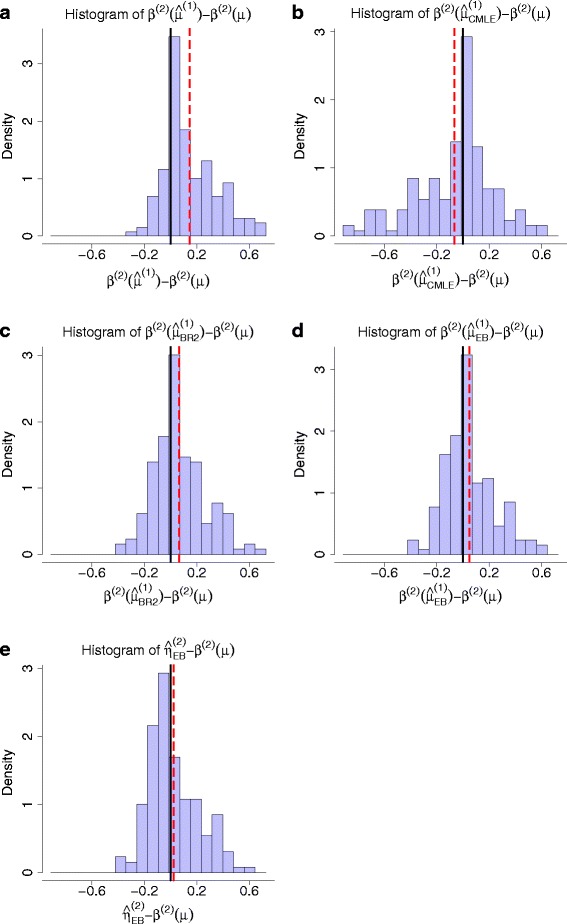
The histograms of the error in power estimation for all associated SNPs identified from primary study. **a** The replication study’s power is estimated by plugging the observed effect size in power calculation formula, i.e. $\beta ^{(2)}\left (\widehat {\mu }^{(1)}\right)$. **b** The power is estimated by plugging the CMLE based corrected effect size $\widehat {\mu }^{(1)}_{\textit {CMLE}}$ in power calculation formula, i.e. $\beta ^{(2)}\left (\widehat {\mu }^{(1)}_{\textit {CMLE}}\right)$. **c** The power is estimated by plugging in the BR2 estimator, i.e. $\beta ^{(2)}\left (\hat {\mu }^{(1)}_{BR2}\right)$. **d** The power is estimated by plugging the EB based corrected effect size $\widehat {\mu }^{(1)}_{\textit {EB}}$ in power calculation, i.e. $\beta ^{(2)}\left (\widehat {\mu }^{(1)}_{\textit {EB}}\right)$. **e** The power is estimated by EB based method, i.e. $\widehat {\eta }^{(2)}_{\textit {EB}}$. The mean value of the estimation error is drawn with vertical dashed line. From the figure, it can be seen that $\widehat {\eta }^{(2)}_{\textit {EB}}$ has the smallest bias in power estimation. The biases for these 5 estimators are 0.144, -0.068, 0.045, 0.047 and 0.021, respectively

In order to check whether our EB based power estimator $\widehat {\eta }_{\textit {EB}}^{(2)}$ can overcome the winner’s curse, the histogram of the differences between estimated values and true values is shown. As a comparison, we will show the corresponding histogram for power estimator by plugging in observed effect size first. A lot of methods have been proposed to overcome the winner’s curse in terms of effect size estimation. CMLE and EB can be used directly in *z*-values of *log*-odds ratio test. The individual-level genotype data are also simulated so that bootstrap based bias reduction method BR2 [10] can also be used as a comparison (We modified the implementation code of BR2 so that *log*-odds ratio test can be used in the software). A direct thinking is to plug these adjusted estimators in the power calculation formula. The corresponding histograms for these three adjusted plug-in based estimators are shown as comparisons. In Fig. 3a, we use plug-in rule to estimate the replication study’s power, where the observed effect size is plugged in. The estimated power is $\beta ^{(2)}\left (\widehat {\mu }^{(1)}\right)$. We plot the histogram of the difference between $\beta ^{(2)}\left (\widehat {\mu }^{(1)}\right)$ and the true power values *β*^(2)^(*μ*) in the figure. The overestimated effect size makes the estimated replication study’s power overestimated as well. Figure 3b plots the histogram of the difference between $\beta ^{(2)}\left (\widehat {\mu }^{(1)}_{\textit {CMLE}}\right)$ and *β*^(2)^(*μ*). The winner’s curse has disappeared, but there is a large downward bias in the estimated results. Equation (10) also introduces an Empirical Bayes estimator of the effect size, which reads

(19) $$ \widehat{\mu}^{(1)}_{EB}=\frac{1}{1+\left(\sigma^{(1)}/\widehat{\sigma}_{0}\right)^{2}} \widehat{\mu}^{(1)}.  $$

Figure 3c, d plot the histogram of $\beta ^{(2)}\left (\widehat {\mu }^{(1)}_{BR2}\right)-\beta ^{(2)}(\mu)$ and $\beta ^{(2)}\left (\widehat {\mu }^{(1)}_{\textit {EB}}\right)-\beta ^{(2)}(\mu)$, respectively. Large upward biases still exist in the histograms. In contrast, Fig. 3e plots the histogram of $\widehat {\eta }^{(2)}_{\textit {EB}}-\beta ^{(2)}(\mu)$, where $\widehat {\eta }^{(2)}_{\textit {EB}}$ is our proposed EB based power estimator. The bias almost disappeared, indicating $\widehat {\eta }^{(2)}_{\textit {EB}}$ is better than other estimators of the replication study’s power in terms of overcoming the winner’s curse. The experiment has run 5 times, and the same conclusion holds in each run. The empirical biases of these five estimators can be seen in the Table 2.

**Table 2:** Empirical biases of power estimators of the replication study in the simulation experiments. The settings of the experiments can be seen in the main text

	$\beta ^{(2)}\left (\widehat {\mu }^{(1)}\right)$	$\beta ^{(2)}\left (\widehat {\mu }_{\textit {CMLE}}^{(1)}\right)$	$\beta ^{(2)}\left (\widehat {\mu }_{BR2}^{(1)}\right)$	$\beta ^{(2)}\left (\widehat {\mu }_{\textit {EB}}^{(1)}\right)$	$\widehat {\eta }_{\textit {EB}}^{(2)}$
Run 1	0.142	−0.113	0.038	0.058	**0.032**
Run 2	0.146	−0.109	0.045	0.021	**0.001**
Run 3	0.144	−0.068	0.045	0.047	**0.021**
Run 4	0.137	−0.090	0.042	0.052	**0.026**
Run 5	0.144	−0.126	0.026	0.038	**0.016**
Average	0.142	−0.101	0.039	0.043	**0.019**

$\beta ^{(2)}\left (\widehat {\mu }^{(1)}\right)$, $\beta ^{(2)}\left (\widehat {\mu }_{\textit {CMLE}}^{(1)}\right)$, $\beta ^{(2)}\left (\widehat {\mu }_{BR2}^{(1)}\right)$ and $\beta ^{(2)}\left (\widehat {\mu }_{\textit {EB}}^{(1)}\right)$ are the plug-in based estimators by using observed effect size, CMLE, BR2 and EB in the effect size estimation. $\widehat {\eta }_{\textit {EB}}^{(2)}$ is proposed EB-based estimator. Bold face indicates the estimator achieving the smallest bias. In the experiments, $\widehat {\eta }_{\textit {EB}}^{(2)}$ behaves better than others in terms of bias reduction

Table 3 shows the root mean square error (RMSE) of the five estimators $\beta ^{(2)}\left (\widehat {\mu }^{(1)}\right)$, $\beta ^{(2)}\left (\widehat {\mu }_{\textit {CMLE}}^{(1)}\right)$, $\beta ^{(2)}\left (\widehat {\mu }_{BR2}^{(1)}\right)$, $\beta ^{(2)}\left (\widehat {\mu }_{\textit {EB}}^{(1)}\right)$ and $\widehat {\eta }^{(2)}_{\textit {EB}}$ in the 5 runs. We can see that $\widehat {\eta }^{(2)}_{\textit {EB}}$ is better than other methods in terms of estimation accuracy.

**Table 3:** Root mean square error (RMSE) of power estimators of the replication study in the simulation experiments. The settings of the experiments can be seen in the main text

	$\beta ^{(2)}\left (\widehat {\mu }^{(1)}\right)$	$\beta ^{(2)}\left (\widehat {\mu }_{\textit {CMLE}}^{(1)}\right)$	$\beta ^{(2)}\left (\widehat {\mu }_{BR2}^{(1)}\right)$	$\beta ^{(2)}\left (\widehat {\mu }_{\textit {EB}}^{(1)}\right)$	$\widehat {\eta }_{\textit {EB}}^{(2)}$
Run 1	0.246	0.334	0.201	0.202	**0.195**
Run 2	0.243	0.312	0.196	0.191	**0.188**
Run 3	0.247	0.303	0.203	0.198	**0.192**
Run 4	0.236	0.307	0.186	0.192	**0.186**
Run 5	0.249	0.317	0.198	0.196	**0.194**
Average	0.244	0.315	0.197	0.196	**0.191**

$\beta ^{(2)}\left (\widehat {\mu }^{(1)}\right)$, $\beta ^{(2)}\left (\widehat {\mu }_{\textit {CMLE}}^{(1)}\right)$, $\beta ^{(2)}\left (\hat {\mu }^{(1)}_{BR2}\right)$ and $\beta ^{(2)}\left (\widehat {\mu }_{\textit {EB}}^{(1)}\right)$ are the plug-in based estimators by using observed effect size, CMLE, BR2 and EB in the effect size estimation. $\widehat {\eta }_{\textit {EB}}^{(2)}$ is proposed EB-based estimator. Bold face indicates the estimator achieving the smallest RMSE. In the experiments, $\widehat {\eta }_{\textit {EB}}^{(2)}$ behaves better than others in terms of higher estimation accuracy

To investigate the performance of the interval estimation, the coverage probability of 95 *%* credible intervals for all identified associated SNPs is shown in Table 4. The coverage probabilities by using pure Monte-carlo sampling are presented on the left side. In agreement with our analysis in the last section, the coverage probability is lower than the nominal value 95 *%*. The coverage probabilities by using modified method which combines Monte-carlo sampling and bootstrap are shown on the right side, which are closer to the nominal value. The credible interval given by the combined method is well-calibrated.

**Table 4:** Coverage probability of the 95 *%* credible intervals in simulation experiments. The simulation settings can be seen in the main text

	Without Bootstrap	With Bootstrap
Run 1	0.932	0.960
Run 2	0.947	0.960
Run 3	0.918	0.943
Run 4	0.914	0.949
Run 5	0.878	0.925
Average	0.918	0.947

Column “No bootstrap” is the coverage probability of the 95 *%* credible intervals created by using pure Monte Carlo method. Column “Bootstrap” is the coverage probability of the 95 *%* credible intervals created by using the combined method of Monte Carlo sampling and bootstrap. From the experiments’ results, the coverage probability of combined method is closer to the nominal value

**Fig. 4:**
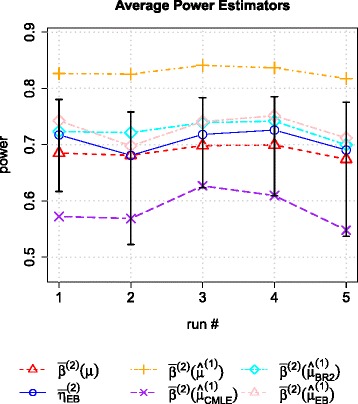
The true value of average power is covered by the credible interval in simulation experiments. The EB based average power estimator $\bar {\eta }^{(2)}_{\textit {EB}}$ estimates more accurately than other plug-in based average power estimators $\bar {\beta }^{(2)}\left (\widehat {\mu }^{(1)}\right)$, $\bar {\beta }^{(2)}\left (\widehat {\mu }_{\textit {CMLE}}^{(1)}\right)$, $\bar {\beta }^{(2)}\left (\hat {\mu }^{(1)}_{BR2}\right)$ and $\bar {\beta }^{(2)}\left (\widehat {\mu }_{\textit {EB}}^{(1)}\right)$. Average power $\bar {\beta }^{(2)}(\mu)$ is defined in the main text. A method combining Monte Carlo method and bootstrap is proposed to create the credible intervals of the replication study’s average power. In the 5 runs of the simulation experiments, the underlying true values of the average power is covered by created intervals. Compared to other plug-in based average power estimators, $\bar {\eta }^{(2)}_{\textit {EB}}$ is the closest to $\bar {\beta }^{(2)}(\mu)$

Figure 4 shows the average powers and their estimated results in the 5 runs. The true value of the average power is covered by 95 % credible interval in all runs. For comparison, we also show the estimated average power by using plug-in rule, i.e. $\bar {\beta }^{(2)}\left (\widehat {\mu }^{(1)}\right)$, $\bar {\beta }^{(2)}\left (\widehat {\mu }^{(1)}_{\textit {CMLE}}\right)$, $\beta ^{(2)}\left (\widehat {\mu }_{BR2}^{(1)}\right)$ and $\bar {\beta }^{(2)}\left (\widehat {\mu }^{(1)}_{\textit {EB}}\right)$, in each run. The estimated power for each association is also weighted by its local true discovery rate *ltdr* in each average power estimator. The figure shows that the EB based average power estimator $\bar {\eta }^{(2)}_{\textit {EB}}$ is much closer to the true value $\bar {\beta }^{(2)}(\mu)$. These results indicate that the estimated average power can be regarded as a proxy of the average power, which can be used to design replication study.

To check the performance of our method when the effect sizes of the associated SNPs are do not follow normal distribution, we also simulated data with the following distributed effect sizes:

(20) $$ \mu \sim 0.9\delta_{0}+0.1t_{5,0.2}   $$

and

(21) $$ \mu \sim 0.9\delta_{0}+0.07N(0,0.04)+0.03N(0,0.16),   $$

where *t*_5,0.02_ is a scaled *t* distribution with degree of freedom 5 and scaling factor 0.2. The distribution of the associated SNPs’ effect sizes follow the Gaussian mixture model in the second case. The average empirical biases and RMSE of all estimators in these two cases are shown in Tables 5 and 6, respectively. From the tables, we can see that our method is still better in terms of overcoming winner’s curse and providing higher estimation accuracy.

**Table 5:** When effect sizes follow the distribution of Eq. (20), the average empirical bias and root mean square error (RMSE) of power estimators of the replication study in the simulation experiments

Average	$\beta ^{(2)}\left (\widehat {\mu }^{(1)}\right)$	$\beta ^{(2)}\left (\widehat {\mu }_{\textit {CMLE}}^{(1)}\right)$	$\beta ^{(2)}\left (\widehat {\mu }_{BR2}^{(1)}\right)$	$\beta ^{(2)}\left (\widehat {\mu }_{\textit {EB}}^{(1)}\right)$	$\widehat {\eta }_{\textit {EB}}^{(2)}$
Empirical Bias	0.085	−0.079	0.023	0.028	**0.003**
RMSE	0.189	0.279	0.167	0.168	**0.163**

Bold face indicates the estimator achieving the smallest value in terms of bias or RMSE. In the experiments, $\widehat {\eta }_{\textit {EB}}^{(2)}$ behaves better than others in terms of bias reduction and providing high estimation accuracy

**Table 6:** When effect sizes follow the distribution of Eq. (21), the average empirical bias and root mean square error (RMSE) of power estimators of the replication study in the simulation experiments

Average	$\beta ^{(2)}\left (\widehat {\mu }^{(1)}\right)$	$\beta ^{(2)}\left (\widehat {\mu }_{\textit {CMLE}}^{(1)}\right)$	$\beta ^{(2)}\left (\widehat {\mu }_{BR2}^{(1)}\right)$	$\beta ^{(2)}\left (\widehat {\mu }_{\textit {EB}}^{(1)}\right)$	$\widehat {\eta }_{\textit {EB}}^{(2)}$
Empirical Bias	0.071	−0.081	0.015	0.033	**0.007**
RMSE	0.173	0.263	0.153	0.154	**0.150**

Bold face indicates the estimator achieving the smallest value in terms of bias or RMSE. $\widehat {\eta }_{\textit {EB}}^{(2)}$ behaves better than others in terms of bias reduction and providing high estimation accuracy in the experiments

### WTCCC datasets

To give an application example of our proposed method, we will determine the sample size of replication study used for verifying the 6 human common diseases’ findings from Wellcome Trust Case Control Consortium (WTCCC). The 6 diseases include coronary artery disease, Crohn’s disease, hypertension, rheumatoid arthritis, type 1 diabetes and type 2 diabetes. Each disease has 2000 cases in the dataset of the primary study. There are 3000 shared controls among all datasets. The following quality control procedure is used in the primary study’s datasets:

1. Missing data control: Chiamo score is used as genotype calling accuracy in the WTCCC data. The genotypes with Chiamo score <0.95 are regarded as missing values. The SNPs with more than 10 % missing entries are removed.
2. Minor allele frequency control: Among all samples, the SNPs with minor allele frequency <0.05 are removed.
3. Hardy-Weinberg equilibrium control: The SNPs with *p*-values <0.001 in the Hardy-Weinberg equilibrium test are removed.

**Fig. 5:**
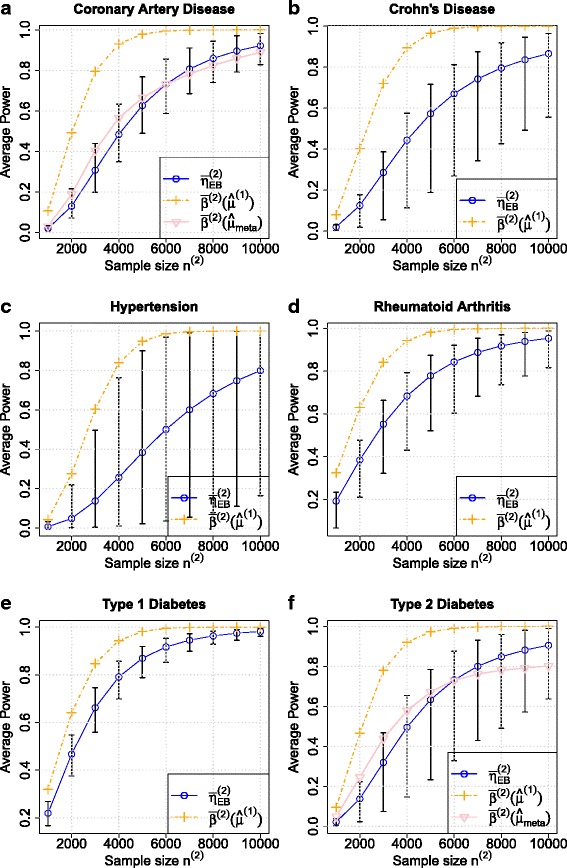
The relationship between estimated average power $\bar {\eta }_{\textit {EB}}^{(2)}$ and the sample size of the replication study *n* ^(2)^ for 6 diseases of the WTCCC dataset: **a** coronary artery disease, **b** Crohn’s disease, **c** hypertension, **d** rheumatoid arthritis, **e** type 1 diabetes, **f** type 2 diabetes. The Control-to-Case ratio of the replication study is set to 1. The significance levels used in the primary study and the replication study are *α*_1_=5×10^−8^ and *α*_2_=5×10^−6^, respectively. As a comparison, the relationship between $\bar {\beta }^{(2)}\left (\hat {\mu }^{(1)}\right)$ and *n* ^(2)^ are also shown in the figure. For a fixed *n* ^(2)^, $\bar {\beta }^{(2)}\left (\hat {\mu }^{(1)}\right)$ is much larger than $\bar {\eta }_{\textit {EB}}^{(2)}$. In (**a**) and (**f**), $\bar {\beta }^{(2)}\left (\hat {\mu }_{\textit {meta}}\right)$ is the average power estimator by plugging the *log*-odds ratio obtained from high power meta-analysis study: CARDIoGRAM GWAS [22] and DIAGRAM GWAS [23], respectively. It can be shown that $\bar {\eta }_{\textit {EB}}^{(2)}$ is close to $\bar {\beta }^{(2)}\left (\hat {\mu }_{\textit {meta}}\right)$

The significance levels used in primary study and replication study are *α*_1_=5×10^−8^ and *α*_2_=5×10^−6^, respectively. The Control-to-Case ratio of the replication study is set to 1. The inferred hyperparameters *π*_0_ and ${\sigma _{0}^{2}}$ can be seen in Table 7. With these parameters, the relationships between the estimated average power using EB based method $\bar {\eta }^{(2)}_{\textit {EB}}$ and sample size of replication study *n*^(2)^ can be seen in Fig. 5. In conclusion, to achieve 80 % average power of the replication study, we will need 6885 individuals for coronary artery disease, 8092 individuals for Crohn’s disease, 10,014 individuals for hypertension, 5291 individuals for rheumatoid arthritis, 4094 individuals for type 1 diabetes and 6988 individuals for type 2 diabetes. The detail about the sample sizes needed for different values of average power can be seen in Table 8.

**Table 7:** The estimated hyperparameters *π*_0_ and ${\sigma _{0}^{2}}$ for 6 diseases of WTCCC dataset

	$\widehat {\pi }_{0}$	$\widehat {\sigma }_{0}^{2}$
Coronary artery disease	0.949	0.004
Crohn’s disease	0.840	0.006
Hypertension	0.966	0.007
Rheumatoid arthritis	0.947	0.008
Type 1 diabetes	0.967	0.014
Type 2 diabetes	0.940	0.005

*π*_0_ is the proportion of true null hypotheses among all SNPs. ${\sigma _{0}^{2}}$ is the variance of the effect sizes among all associated SNPs

**Table 8:** Sample size of the replication study needed for 6 diseases of WTCCC dataset when average power is estimated by EB based method. The Control-to-Case ratio of the replication study is set to 1. The significance levels used in the primary study and the replication study are *α*_1_=5×10^−8^ and *α*_2_=5×10^−6^, respectively

	50 %	60 %	70 %	80 %	90 %
Coronary artery disease	4095	4784	5652	6885	9121
Crohn’s disease	4405	5252	6376	8092	11,552
Hypertension	5993	6992	8244	10,014	13,215
Rheumatoid arthritis	2666	3329	4147	5291	7357
Type 1 diabetes	2146	2640	3249	4094	5588
Type 2 diabetes	4027	4726	5633	6988	9721

As a comparison, we also plot relationships between $\bar {\beta }^{(2)}\left (\widehat {\mu }^{(1)}\right)$ and *n*^(2)^ in Fig. 5, where $\bar {\beta }^{(2)}\left (\widehat {\mu }^{(1)}\right)$ is the estimated average power by plugging in observed effect sizes. For a given sample size *n*^(2)^, the estimated average power value using EB based method is much smaller than $\bar {\beta }^{(2)}\left (\widehat {\mu }^{(1)}\right)$. This is reasonable because $\bar {\beta }^{(2)}\left (\widehat {\mu }^{(1)}\right)$ is overestimated due to the winner’s curse, which is alleviated in the EB based method. To achieve 80 % average power of the replication study, the sample size needed is 3023 for coronary artery disease, 3369 for Crohn’s disease, 3788 for hypertension, 2748 for rheumatoid arthritis, 2706 for type 1 diabetes and 3095 for type 2 diabetes when using $\bar {\beta }^{(2)}\left (\widehat {\mu }^{(1)}\right)$ as the estimator of average power. The sample sizes needed for other values of average power are listed in Table 9. These determined sample sizes are much smaller than the sample sizes determined by EB based method, indicating an underpowered study will be designed if we estimate power with observed effect sizes.

**Table 9:** Sample size of the replication study needed for 6 diseases of WTCCC dataset when average power is estimated by plugging in observed effect sizes. The Control-to-Case ratio of the replication study is set to 1. The significance levels used in the primary study and the replication study are *α*_1_=5×10^−8^ and *α*_2_=5×10^−6^, respectively

	50 %	60 %	70 %	80 %	90 %
Coronary artery disease	2019	2290	2608	3023	3675
Crohn’s disease	2270	2572	2922	3369	4058
Hypertension	2672	2988	3345	3788	4448
Rheumatoid arthritis	1553	1892	2273	2748	3465
Type 1 diabetes	1532	1856	2229	2706	3443
Type 2 diabetes	2085	2357	2676	3095	3775

For coronary artery disease and type 2 diabetes, we obtained the publicly available summary statistics of the meta-analysis from two consortiums: CARDIoGRAMplusC4D Consortium [22] and DIAGRAM Consortium [23], respectively. CARDIoGRAM GWAS is a meta-analysis of 22 GWAS studies of European descent involving 22,233 cases and 64,762 controls. The odds ratio calculated from high power CARDIoGRAM GWAS will be used as underlying true odds ratio to calculate the average power of the replication study for coronary artery disease in WTCCC. The average power obtained in this manner is denoted as $\bar {\beta }^{(2)}\left (\hat {\mu }_{\textit {meta}}\right)$. Figure 5a plots the relationship between $\bar {\beta }^{(2)}\left (\hat {\mu }_{\textit {meta}}\right)$ and *n*^(2)^, which is the sample size needed in the replication study. The figure shows that our EB based power estimator $\bar {\eta }_{\textit {EB}}^{(2)}$ is very close to the power calculated using the results of CARDIoGRAM GWAS. Also it can be shown that $\bar {\beta }^{(2)}\left (\hat {\mu }_{\textit {meta}}\right)$ is in the credible interval we estimated. DIAGRAM GWAS is a meta-analysis consisting of 12,171 type 2 diabetes cases and 56,862 controls across 12 GWAS from European descent populations. Similar to CARDIoGRAM GWAS, the allele based odds ratio calculated from DIAGRAM GWAS is used for calculating the average power of the replication study for type 2 diabetes in WTCCC. Figure 5f plots the relationship between $\bar {\beta }^{(2)}\left (\hat {\mu }_{\textit {meta}}\right)$ and *n*^(2)^. It can be shown that the result estimated by our EB based method $\bar {\eta }_{\textit {EB}}^{(2)}$ is close to the power calculated using the results of DIAGRAM GWAS.

If the values of the local true discovery rates *ltdr*^(1)^ have nearly the same level for all identified associations in the primary study, the variance of the average power will be inversely proportional to the number of the associations. When the identified number is small in the primary study, the credible interval for the average power is rather wide. This can be illustrated in the study of hypertension, where there is only 1 association showing genome-wide significance. From Fig. 5c, we can see that the credible interval is rather wide. If we want to consider the credible interval for this situation, then the sample size can drastically increased.

### Discussion

We propose to design replication study under the case-control setting where *log*-odds ratio test is used. The method can also be generalized to other tests within *z*-test scheme, such as regression slope test used for quantitative trait.

As described in [7], the winner’s curse depends strongly on the power of primary study. For a high power primary study, most non-null SNPs will result in significant associations after random draws from the population. Hence, the bias will be small in this case. There are more and more high power studies conducted for common diseases by using pooling strategy or meta-analysis strategy, but the high power studies for rare diseases are limited. Hence, it is still helpful and necessary to propose a designing procedure for the replication study with the consideration of winner’s curse.

With the development of the cost-effective sequencing technique, the targets of association studies extend from common variations to rare variants. A commonly used strategy to discover associations with rare variants is the collapsing method [24], in which several rare variants in a certain group are pooled together to enrich the signal. For each group, a “super variant” is constructed. If *log*-odds ratio test is adopted in testing the association between “super variant” and the disease, our method can be used directly for designing the replication study.

Some limitations of our approach need to be mentioned.

1. The assumption of our approach is that all SNPs’ effect sizes are drawn independently from a two-component mixture distribution. Linkage disequilibrium widely exists in SNPs. Correlated genotype patterns can also introduce correlation between their effect sizes. The power estimation can be further improved by using correlation information in the prior set-up.
2. Our proposed method assumes the effect sizes of associated SNPs are normally distributed. This thin tail distribution may not be realistic. How to design of replication study with other heavy-tail prior needs to be discussed.

## Conclusions

Replication study is commonly used to verify findings discovered from GWAS. Power analysis is essential in designing a replication study. Traditional approach will not extract information from primary study. Also it will need users to specify a parameter *μ*_*min*_, which is subjective. Power estimation approach may address this problem, but there are several challenges in power estimation: the winner’s curse, credible interval and summarization.

In this paper, we propose an EB based power estimation method to resolve these challenges. Simulation experiments show our approach is better than other plug-in based approaches in terms of overcoming the winner’s curse and providing higher estimation accuracy. We also use simulation experiments to demonstrate the well calibration of the constructed credible interval. As an application example, we use our approach to determine the sample size needed in the WTCCC datasets of 6 diseases. Our approach gives an objective way to design replication study using information extracted from primary study.

## Appendix

### Appendix 1 — *η*^(2)^ is the minimizer of $R(\widehat {\theta })$

The Bayes risk $R(\widehat {\theta })$ can be derived as follows:

(22) $$  {\fontsize{8.5}{6}\begin{aligned} R(\widehat{\theta}) &= E_{\mu, z^{(1)}}\left(\left(\widehat{\theta}-\beta^{(2)}(\mu)\right)^{2}\big| |Z^{(1)}|>z_{\alpha_{1}/2}, \mathcal{H}_{1}\right) \\ &= \int_{-\infty}^{\infty}\! \left[\int_{|z^{(1)}|>z_{\alpha_{1}/2}}\!\left(\widehat{\theta}-\beta^{(2)}(\mu)\right)^{2} \!\frac{p\left(z^{(1)}|\mu\right)}{P\left(|Z^{(1)}|>z_{\alpha_{1}/2}\big| \mu\right)}d z^{(1)} \right]\\ &\quad\times p\left(\mu \big| |Z^{(1)}|>z_{\alpha_{1}/2}, \mathcal{H}_{1}\right) d\mu \\ &= \frac{1}{P\left(Z^{(1)}>z_{\alpha_{1}/2}\big| \mathcal{H}_{1}\right)} \int_{-\infty}^{\infty} \left[\int_{|z^{(1)}|>z_{\alpha_{1}/2}}\left(\widehat{\theta}-\beta^{(2)}(\mu)\right)^{2} \right.\\ &\quad \times\left. \vphantom{\int_{|z^{(1)}|>z_{\alpha_{1}/2}}}p\left(z^{(1)}\big|\mu\right)dz^{(1)} \right] p\left(\mu\big| \mathcal{H}_{1}\right)d\mu \\ &= \frac{1}{P\left(Z^{(1)}>z_{\alpha_{1}/2}\big|\mathcal{H}_{1}\right)} \int_{|z^{(1)}|>z_{\alpha_{1}/2}} \left[ \int_{-\infty}^{\infty}\left(\widehat{\theta}-\beta^{(2)}(\mu)\right)^{2}\right.\\ &\quad\times \left. \vphantom{\int_{-\infty}^{\infty}}p\left(\mu \big|z^{(1)}, \mathcal{H}_{1}\right)d\mu \right] p\left(z^{(1)}\right)dz^{(1)}. \\ \end{aligned}}  $$

The last equality is hold by Fubini’s theorem.

From the last equality, it can be seen that the Bayesian predictive power *η*^(2)^ is the minimizer of the expression in the brace for each value of *z*^(1)^. Hence *η*^(2)^ is also the minimizer of $R(\widehat {\theta })$.

### Appendix 2 — Derivation of *η*^(2)^ under two-component mixture prior

The following property of multivariate Gaussian distribution is proved in the Chapter 2 of [25], which can be used to derive *η*^(2)^.

#### **Property1**.

If **Z**|**μ**∼*N*_*p*_(**μ**,**Σ**), and **μ**∼*N*_*p*_(*μ*_0_,*Σ*_0_), then

(23) $$\begin{array}{@{}rcl@{}} \mathbf{Z}\sim N_{p}\left(\mathbf{\mu_{0}}, \mathbf{\Sigma}+\mathbf{\Sigma_{0}}\right)~\text{and}~ \mathbf{\mu} \big| \mathbf{z}&\sim& N_{p}\left(\mathbf{W\mu_{0}+(I-W)z},\right.\\ &&\left. \mathbf{(I-W)\Sigma}\right) \end{array} $$

where **W**=*Σ*(*Σ*_0_+*Σ*)^−1^

Because *z*^(1)^∼*N*(*μ*/*σ*^(1)^,1) and $\left (\mu \big | \mathcal {H}_{1}\right) \sim N\left (0, {\sigma _{0}^{2}}\right)$, the following can be obtained by using Property 1:

(24) $$ \left(\mu \big| z^{(1)},\mathcal{H}_{1}\right)\sim N\left(\lambda \hat{\mu}^{(1)},\lambda \left(\sigma^{(1)}\right)^{2}\right),  $$

where $\lambda =\frac {1}{1+\left (\sigma ^{(1)}/\sigma _{0}\right)^{2}}$ is a shrinkage effect factor. Under $\mathcal {H}_{1}$, the posterior distribution of *Z*^(2)^ is

(25) $$ \left(Z^{(2)} \big| z^{(1)},\mathcal{H}_{1}\right)\!\sim\! N\left(z^{*}\,=\,\lambda \frac{\hat{\mu}^{(1)}}{\sigma^{(2)}},\! \left(\sigma^{*}\right)^{2}\,=\,1\,+\,\lambda\left(\frac{\sigma^{(1)}}{\sigma^{(2)}}\right)^{2} \right).  $$

Then the Bayesian predictive power of the replication study reads:

(26) $$ \eta^{(2)}= \Phi\left(\frac{sgn\left(z^{(1)}\right)z^{*}-z_{\alpha_{2}}}{\sigma^{*}}\right),  $$

where *Φ*(*x*) is the cumulative density function (cdf) of *N*(0,1).

### Appendix 3 — Derivation of the ${\sigma _{0}^{2}}$ estimator

By using Property 1, the marginal distribution of *Z*^(1)^ is

(27) $$ Z^{(1)}\sim \pi_{0} N(0,1)+(1-\pi_{0}) N\left(0, 1+\left(\frac{\sigma_{0}}{\sigma^{(1)}}\right)^{2}\right),   $$

which is a two-component Gaussian mixture model. Hence, the squared of *Z*^(1)^ is distributed as

(28) $$ \left(Z^{(1)}\right)^{2}\sim \pi_{0} {\chi_{1}^{2}}+(1-\pi_{0}) \left(1+\left(\frac{\sigma_{0}}{\sigma^{(1)}}\right)^{2}\right){\chi_{1}^{2}},  $$

where ${\chi _{1}^{2}}$ is the 1 degree of freedom *χ*^2^ distribution. The expectation reads

(29) $$ E\left(\left(Z^{(1)}\right)^{2}\right)=\pi_{0}+(1-\pi_{0}) \left(1+\left(\frac{\sigma_{0}}{\sigma^{(1)}}\right)^{2}\right).  $$

By summing over the test statistics of all SNPs, we can obtain

(30) $$  E\left(\sum_{i=1}^{m} \left(Z_{i}^{(1)}\right)^{2}\right)\,=\,m\pi_{0}+\left(1-\pi_{0}\right)\!\! \left(m\,+\,{\sigma_{0}^{2}}\sum_{i=1}^{m}\! \left(1/\sigma^{(1)}_{i}\right)^{2}\!\right)\!,  $$

which introduce an estimator of ${\sigma _{0}^{2}}$

(31) $$ \widehat{\sigma}_{0}^{2}=\left(\frac{\sum_{i=1}^{m} \left(z^{(1)}_{i}\right)^{2}-m\pi_{0}}{\left(1-\pi_{0}\right)}-m\right)/\sum_{i=1}^{m} \left(1/\sigma^{(1)}_{i}\right)^{2}.  $$

### Appendix 4 — Derivation of *ltdr*^(1)^ under a two-component mixture prior

With Eq. (27), the local true discovery rate of the primary study reads:

(32) $$ ltdr^{(1)}= \frac{\pi_{1} \phi \left(z^{(1)} /\sqrt{1+\left(\widehat{\sigma}_{0}/\sigma^{(1)}\right)^{2}}\right)}{\pi_{0} \phi(z^{(1)}) +\pi_{1} \phi \left(z^{(1)} /\sqrt{1+\left(\widehat{\sigma}_{0}/\sigma^{(1)}\right)^{2}}\right)},  $$

where *ϕ*(*x*) is the probability density function (pdf) of *N*(0,1).

## References

[1] Hindorff LA, MacArthur J, Morales J, Junkins HA, Hall PN, Klemm AK, et al. A catalog of published genome-wide association studies. Available at: http://www.genome.gov/gwastudies/. Accessed [2015.05.28] (Web Resources).

[2] Balding DJ (2006). A tutorial on statistical methods for population association studies. Nat Rev Genet.

[3] NCI-NHGRI Working Group on Replication in Association Studies. Replicating genotype–phenotype associations. Nature. 2007; 447(7145):655–60. 10.1038/447655a 17554299

[4] Kraft P, Zeggini E, Ioannidis JP (2009). Replication in genome-wide association studies. Stat Sci Rev J Inst Math Stat.

[5] Ioannidis JP (2008). Why most discovered true associations are inflated. Epidemiology.

[6] Göring HH, Terwilliger JD, Blangero J (2001). Large upward bias in estimation of locus-specific effects from genomewide scans. Am J Hum Genet.

[7] Zöllner S, Pritchard JK (2007). Overcoming the winner’s curse: estimating penetrance parameters from case-control data. Am J Hum Genet.

[8] Zhong H, Prentice RL (2008). Bias-reduced estimators and confidence intervals for odds ratios in genome-wide association studies. Biostatistics.

[9] Ghosh A, Zou F, Wright FA (2008). Estimating odds ratios in genome scans: an approximate conditional likelihood approach. Am J Hum Genet.

[10] Sun L, Dimitromanolakis A, Faye LL, Paterson AD, Waggott D, Bull SB (2011). The DCCT/EDIC Research Group.: BR-squared: a practical solution to the winner’s curse in genome-wide scans. Human genetics.

[11] Xu L, Craiu RV, Sun L (2011). Bayesian methods to overcome the winner’s curse in genetic studies. Ann Appl Stat.

[12] Ferguson JP, Cho JH, Yang C, Zhao H (2013). Empirical Bayes correction for the winner’s curse in genetic association studies. Genet Epidemiol.

[13] Wellcome Trust Case Control Consortium. Genome-wide association study of 14,000 cases of seven common diseases and 3,000 shared controls. Nature. 2007; 447(7145):661–78. 10.1038/nature05911 PMC2719288 17554300

[14] Woolf B (1955). On estimating the relation between blood group and disease. Ann Hum Genet.

[15] Lecoutre B (2001). Bayesian predictive procedure for designing and monitoring experiments. Bayesian Methods with Applications to Science, Policy and Official Statistics.

[16] Yang J, Benyamin B, McEvoy BP, Gordon S, Henders AK, Nyholt DR (2010). Common SNPs explain a large proportion of the heritability for human height. Nat Genet.

[17] Park J-H, Wacholder S, Gail MH, Peters U, Jacobs KB, Chanock SJ (2010). Estimation of effect size distribution from genome-wide association studies and implications for future discoveries. Nat Genet.

[18] Storey JD, Tibshirani R (2003). Statistical significance for genomewide studies. Proc Natl Acad Sci.

[19] Langaas M, Lindqvist BH, Ferkingstad E (2005). Estimating the proportion of true null hypotheses, with application to DNA microarray data. J R Stat Soc Ser B (Stat Methodol).

[20] Jin J, Cai TT (2007). Estimating the null and the proportion of nonnull effects in large-scale multiple comparisons. J Am Stat Assoc.

[21] Efron B. Local false discovery rates. Technical Report 2005-20B. Department of Statistics, Stanford University. 2005.

[22] Mehta NN (2011). Large-scale association analysis identifies 13 new susceptibility loci for coronary artery disease. Circ Cardiovasc Genet.

[23] Morris AP, Voight BF, Teslovich TM, Ferreira T, Segré AV, Steinthorsdottir V (2012). Large-scale association analysis provides insights into the genetic architecture and pathophysiology of type 2 diabetes. Nat Genet.

[24] Bansal V, Libiger O, Torkamani A, Schork NJ (2010). Statistical analysis strategies for association studies involving rare variants. Nat Rev Genet.

[25] Bishop CM (2006). Pattern Recognition and Machine Learning.

