# Erratum to: Power estimation and sample size determination for replication studies of genome-wide association studies

**DOI:** 10.1186/s12864-017-3482-3

**Published:** 2017-01-11

**Authors:** Wei Jiang, Weichuan Yu

**Affiliations:** Department of Electronic and Computer Engineering, The Hong Kong University of Science and Technology, Clear Water Bay, Kowloon, Hong Kong China

## Erratum

In this version of this article that was originally published [1] there were errors:

1. In the online HTML version of the full text, there is an unexpected text “\fontsize8.56” inserted in Eq. (22) of Appendix 1.

2. In the PDF version of the full text, the first paragraph in the right column of Page 3 is overlapped by Eq. (2).

The publisher apologises for these errors.
